# Polymethylmethacrylate Incorporating Nanodiamonds for Denture Repair: *In Vitro* Study on the Mechanical Properties

**DOI:** 10.1055/s-0041-1735792

**Published:** 2021-11-25

**Authors:** Mohammed M. Gad, Mohamed Saber Ali, Ahmad M. Al-Thobity, Yousif A. Al-Dulaijan, Mai El Zayat, Abdel-Naser M. Emam, Sultan Akhtar, Soban Q. Khan, Fahad A. Al-Harbi, Shaimaa M. Fouda

**Affiliations:** 1 Department of Substitutive Dental Sciences, College of Dentistry, Imam Abdulrahman Bin Faisal University, Dammam, Saudi Arabia; 2 Department of Removable Prosthodontics, Faculty of Dental Medicine (Boys), Al-Azhar University, Cairo, Egypt; 3 Department of Biophysics, Institute for Research and Medical Consultations, Imam Abdulrahman Bin Faisal University, Dammam, Saudi Arabia

**Keywords:** nanoparticles, polymethylmethacrylate, reinforcement, repair gap, repair strength

## Abstract

**Objective** This study aimed to evaluate the effect of nanodiamond (ND) addition to repair resin with repair gap modifications on the flexural and impact strength of repaired polymethylmethacrylate denture base.

**Materials and Methods** Heat-polymerized acrylic resin specimens ( *N* = 100/test) were prepared and sectioned to half creating two repair gaps: 2.5- and 0 mm with 45 degrees beveling. They were further divided into subgroups ( *n* = 20) according to ND concentration (control, 0.25%ND, and 0.50%ND), thermocycling (500 cycles) was done to half the specimens in each subgroup. Flexural strength was tested using 3-point bending test and impact strength was tested by Charpy's impact test. Analysis of variance and post-hoc Tukey's tests were performed for data analysis ( *α* = 0.05). Scanning electron microscope was employed for fracture surface analysis and ND distribution.

**Results** Before and after thermocycling, the addition of ND significantly increased the flexural strength and elastic modulus in comparison to control group ( *p* ˂ 0.001), while 0 mm repair gap showed insignificant difference between ND-reinforced groups ( *p* ˃ 0.05). Regarding impact strength, ND addition increased the impact strength with 0 mm gap in comparison to control and 2.5 mm with ND ( *p* ˂0.001), while later groups showed no significant in between ( *p* ˃ 0.05). Comparing thermocycling effect per respective concentration and repair gap, thermocycling adversely affected all tested properties except elastic modulus with 0 mm–0.25 and 0 mm–0.5% and impact strength with 2.5 mm, 2.5 mm–0.25%, 2.5 mm– 0.5% ( *p* ˃ 0.05).

**Conclusion** ND addition combined with decreased repair gap improved the flexural strength, elastic modulus, and impact strength of repaired denture resin, while thermocycling has a negative effect on denture repair strength.

## Introduction

Denture fracture commonly occurs due to the low impact and flexural strength of denture base resin. Sudden drop of the denture is the most common cause of dentures fracture. Denture repair is frequent in dental practice; it requires less expenses and time than fabrication of a new denture. 1 2 The material used for denture repair should attain the original denture color and strength as well as dimensional stability. 3 Autopolymerized acrylic resin is the most commonly used material for denture repair because of its good color match and ease of manipulation that allows chairside repair. 4 However, it has poor strength that ranges between 18 and 81% of heat polymerized acrylic resin. 4 Success of denture repair is affected by the type and/or reinforcement of repair resin, in addition to repair surface treatment and design. 2 5

Denture repair begins with preparing a repair gap to provide space for repair material. 2 The extent of repair gap defines the quantity of added repair material thus affecting the strength of denture repair. 2 5 Several previous studies have tested the size of repair gap, which ranged between 1 and 10 mm. 5 6 7 8 However, limited studies have tested the influence of repair gap size on strength of repaired denture, while most studies have investigated repair design, surface treatment, and reinforcement of repair material. 2 A repair gap of 3 mm or less is preferred due to less repair material used and consequently less polymerization shrinkage and color variation between the denture base and repair resin. 6 7 With decreasing the repair gap from 3 to 1.5 mm, the deflection was decreased by 20%. 8 Gad et al 5 suggested decreasing repair gap even up to 0 mm repair gap and reported an increase in flexural strength as repair gap decreased, while impact strength increased with 2 and 1.5 mm repair gaps. On the other hand, larger repair gaps were affected by thermal cycling. Therefore, decreasing repair gap was recommended as an approach to improve repair strength. 5

Hanna et al 9 reported that 45 degrees bevel repair surface design improved repair strength. Moreover, repair surface treatment with monomer alters the surface structure and improves the bond at repair/resin interface. 9 10 Repair surface beveling with monomer application showed cohesive failure within the repair resin instead of adhesive failure. 2 9 10 The cohesive fracture type of repair resin confirmed the responsibility of repair resin for weak repair strength. 2 Therefore, repair resin reinforcement was suggested using wires, fibers, 2 filler, or nanofillers. 10 11 Nanofillers are widely used due to their inherent properties such as nanoscale with big particular surface area and interfacial interactivity with organic polymers. 12 The main purpose of nanoparticles incorporation into dental polymeric materials was to improve some of the mechanical properties of the final nanocomposites. 12 13

Several nanoparticles were used to improve denture repair material including ZrO _2_ , Al _2_ O _3_ , and SiO _2_ nanoparticles. 10 11 14 15 16 ZrO _2_ nanoparticles addition improved the transverse strength and impact strength of repair resin. 10 11 16 SiO _2_ nanoparticles with 45 degrees beveled repair surface increased flexural strength of repaired acrylic resin. 14 Al _2_ O _3_ nanoparticles improved the flexural strength of repair resin compared with the unmodified resin. 16 Nanodiamond (ND) belongs to nanocarbon family and possesses distinctive properties permitting its use for dental applications. 17 18 19 ND has been tested in previous studies as filler to heat polymerized denture base resin. Al-Harbi et al 18 concluded that the impact strength of polymethylmethacrylate (PMMA) was lowered as ND increased and recommended the addition of ND in low concentrations. Similarly, Protopapa et al 20 reported an improvement in the mechanical properties of provisional restorations fabricated from autopolymerized PMMA resin reinforced with low ND concentrations. Furthermore, addition of ND to PMMA reduced *Candida albicans* adhesion. 19

Although ND showed a positive effect on the properties of PMMA/ND composite, its effect on denture repair base material along with 0 mm repair gap has not been investigated previously. Therefore, the aim of the present study was to determine the influence of low ND concentrations on strength of denture repair resin. The first null hypothesis was that the repair gap variations would not affect the flexural properties and impact strength of autopolymerized repair resin. The second null hypothesis was that addition of ND to autopolymerized repair resin would not affect its flexural and impact strength.

## Materials and Methods

Sample size calculations revealed that 200 specimens (100 per test/ *n* = 10) were required to conduct this study. Acrylic resin specimens were prepared following ISO standard 1567:1999/Amd.1:2003(E). 21 For flexural properties, acrylic plates were prepared in dimensions 65 × 10 × 2.5 mm, while for impact strength specimens were prepared in dimension of 50 mm lengths × 6 mm width × 4 mm thickness. At the middle of impact strength specimen, a standard v-notch was prepared with depth 0.8 mm through the whole 6 mm width of the specimen leaving 3.2 mm thickness below the notch. 21 A customized split press metal mold with required dimensions was used for wax specimens' preparations. Heat polymerized PMAA acrylic resin (Major base 20; Major Prodotti Dentari SPA, Italy) was used to fabricate all specimens following the conventional method for denture base fabrication as described in previous studies. 5 18 A digital caliper was used to evaluate specimens dimension and specimens with improper dimensions were excluded. Approved specimens were kept in distilled water for 2 days at 37°C.

**Fig. 1:**
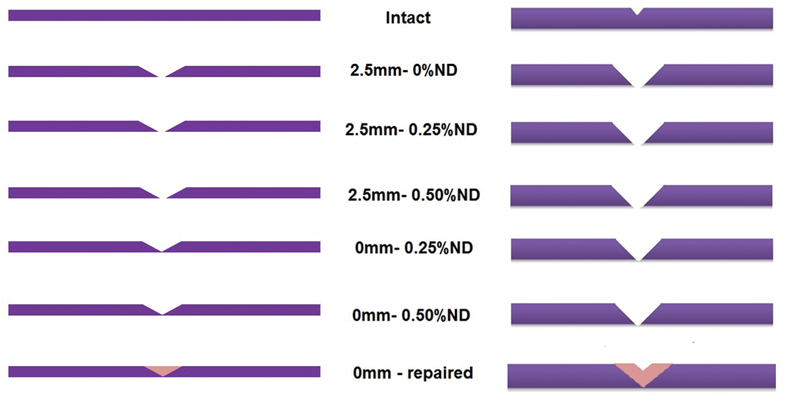
Schematic diagram for acrylic resin specimens preparation and repair. ND, nanodiamond.

Specimens' preparation for repair was performed according to the method described in a previous study. 5 For the 2.5 mm group, a diamond disc (REF 5990 3107, DeguDent GmbH, Wolfgang, Germany) was used to section the specimens creating a repair gap of 2.5 mm and 45°-beveled repair surface. For 0 mm group, the specimens were sectioned into equal halves with 45° bevel at the cameo surface of the inner ends of the two sections preserving the normal length of specimens at on the intaglio side ( Fig. 1 ). 10

ND (Shanghai Richem International Co. Ltd, Shanghai, China) with purity: 98–99%, and particle size: 30–40 nm was treated as described in previous studies. 18 19 Digital scale (S-234, Denver Instrument) was used to weight the treated ND particles in concentrations of 0.25 and 0.50%, by weight of autopolymerized repair PMMA resin powder (Major repair; Prodotti Dentari SPA, Italy). According to ND concentrations and repair gap size, specimens were randomly distributed to five groups: control, without ND addition and 4 tested groups with 0.25% ND, 0.50%ND ( Table 1 ). All mixtures separately were initially mixed with hand and then stirred using electric mixer for 30 minutes at 400 rpm to ensure homogenous distributions of nanoparticles within resin powder. 18

**Table 1:** Specimens grouping and coding according to repair gap, nanodiamond (ND) concentrations, and thermocycling

Thermocycling	Gap	Code	Specifications
0 cycle	2.5 mm	2.5 mm	Repaired with unmodified repair resin
		2.5 mm–0.25%ND	Repaired with repair resin reinforced with 0.25% ND
		2.5 mm–0.50%ND	Repaired with repair resin reinforced with 0.50% ND
	0 mm	0 mm–0.25%ND	Repaired with repair resin reinforced with 0.25% ND
		0 mm–0.50%ND	Repaired with repair resin reinforced with 0.50% ND
5.000 cycles	2.5 mm	2.5 mm	Repaired with unmodified repair resin
		2.5 mm–0.25%ND	Repaired with repair resin reinforced with 0.25% ND
		2.5 mm–0.50%ND	Repaired with repair resin reinforced with 0.50% ND
	0 mm	0 mm–0.25%ND	Repaired with repair resin reinforced with 0.25% ND
		0 mm–0.50%ND	Repaired with repair resin reinforced with 0.50% ND

Treatment of repair surface was done by monomer application for 180 second and then the 2 halves of the specimen were reassembled in the original metal molds. The repair resin was mixed and packed following the manufacturer instructions, overfilling the repair gap, then placed in pot pressure with 45° C. After complete polymerization, specimens were removed from the mold. The excess resin was removed using the conventional finishing and polishing techniques for denture base. Then, specimens' dimensions were reevaluated with the digital caliper and kept in distilled water for 72 hours at 37°C prior testing.

The three-point bending test was performed using a universal testing machine (Instron, 5965, United States). A custom-made stainless steel device that included a 50 mm span between the two supports was employed. Using a 2 mm blunt round end tip, a load with 5 mm/min crosshead speed was performed centrally to the intaglio surface of the specimens at the repaired area. Recording of the maximum load at fracture was performed, and the flexural strength (FS) and elastic modulus were calculated as described in previous studies. 5 21

Charpy's impact testing machine (Digital Charpy Izod impact tester, XJU 5.5, Jinan Hensgrand Instrument Co., Ltd., Jinan, China) was used to measure the impact strength. After horizontal placement of the specimen on a metal jig with 40 mm distance between two supports, a pendulum with 0.5 J weight was fallen at the back side of the specimens (opposite to the notch). The absorbed energy required for specimen fracture was digitally displayed on the monitor and recorded impact strength value (kJ/m ^2^ ). The data were collected and tabulated for statistical analysis.

**Fig. 2:**
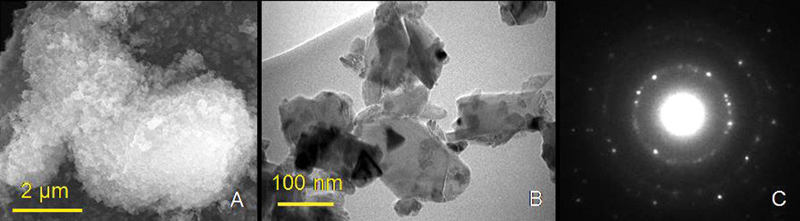
( **A** ) Scanning electron microscopy micrograph of nanodiamond (ND) powder, ( **B** ) transmission electron microscopy image of ND powder, and ( **C** ) corresponding selected area electron diffraction (SAED) pattern for crystalline material.

**Fig. 3:**
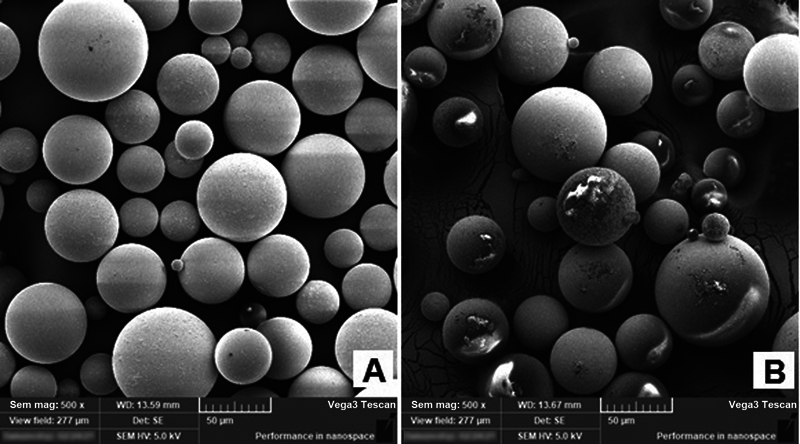
Scanning electron microscopy micrographs of ( **A** ) pure polymethylmethacrylate and ( **B** ) polymethylmethacrylate/nanodiamond mixture.

After testing, the FS specimens were coated with gold and analyzed under scanning electron microscopy (SEM; TESCAN Vega3 with working voltage of 20 kV) as described in previous studies. 18 24 The electron micrographs were recorded at different magnifications, namely x200, x500 and x1000 to reveal the maximum features of the fractured surfaces. To reveal the size, shape, and structure of the individual particles of the nano-diamond (ND), the ND powder (ND dispersion) was deposited onto SEM holder (TEM grid) and examined under SEM and transmission electron microscope (TEM, Morgagni 268, FEI, with working voltage of 80 kV) for high resolution ( Fig. 2A and B ). The crystalline structure of the ND nano-powder was verified by electron diffraction performed in the TEM ( Fig. 2C ). The shape of the ND particles was irregular with thickness of few tens of nanometers. Furthermore, the prepared PMMA/ND mixture was also examined under SEM to realize the distribution and existence of the ND particles within PMMA powder before going to use heat polymerization treatment ( Fig. 3 ).

Statistical package for social sciences (SPSS v. 23) was used to enter and analyzed data. In descriptive statistics, mean and standard deviations were computed. For inferential statistics, normality of the data was tested first by using Shapiro–Wilk test and insignificant results were provided that data was normally distributed; hence, parametric tests were used for analysis. One-way analysis of variance (ANOVA) was used to study the variation in tested properties with different levels of repair gap and with different level of ND concentrations followed by post-hoc Tukey's test for pairwise comparison. Two-way ANOVA was used to study the combined effect of repair gap and thermocycling effect (before and after) on properties. Two independent samples *t* -test was performed to test the significance in difference in averages before and after thermocycling effect for each tested property. Chi-squared test was performed to study the association between thermocycling effect (before and after) and nature of failure. Level of significance was set as 0.05.

## Results

One-way ANOVA was performed to test the effect of variation in repair gap with concentration over the tested properties and it was analyzed separately before and after thermocycling ( Table 2 ). Flexural strength, elastic modulus, and impact strength showed statistically significant differences ( Table 2 ). Flexural strength mean values before thermocycling were significantly higher than that after thermocycling among all the groups ( *p* ˂0.05; ( Table 3 ). The lowest mean value of flexural strength was reported at control group (2.5 mm), while the highest value was recorded with 0.25% ND for both repair gaps (2.5 and 0 mm) before and after thermocycling. Between ND-reinforced groups, insignificant differences were found before thermocycling between 2.5 mm–0.25% versus 0 mm–0.25% ( *p* = 0.505) and 2.5 mm–0.5% versus 0 mm–0.5% ( *p* = 0.664), while for the rest of the pairs, difference was statistically significant. After thermocycling, differences between ND groups were found statistically insignificant in pairs 2.5 mm–0.25% versus 0 mm–0.25% ( *p* = 0.401), 2.5 mm–0.5% versus 0 mm–0.5% ( *p* = 0.305) and 0 mm–0.25% versus 0 mm–0.5% ( *p* = 0.211).

**Table 2:** One-way ANOVA analysis of tested properties before and after thermocycling

Thermocycling	Property	Group	Sum of squares	df	Mean square	F-Value	Sig.
Before	Flexural strength	Between groups	533.688	4	133.422	114.673	0.000 a
		Within groups	23.270	20	1.163		
		Total	556.958	24			
	Elastic modulus	Between groups	1312503.170	4	328125.792	8.098	0.000 a
		Within groups	810387.427	20	40519.371		
		Total	2122890.597	24			
	Impact strength	Between groups	10.844	4	2.711	19.222	0.000 a
		Within groups	2.821	20	0.141		
		Total	13.665	24			
After	Flexural strength	Between groups	270.561	4	67.640	39.464	0.000 a
		Within groups	34.279	20	1.714		
		Total	304.841	24			
	Elastic modulus	Between groups	6170232.621	4	1542558.155	52.281	0.000 a
		Within groups	590099.477	20	29504.974		
		Total	6760332.098	24			
	Impact strength	Between groups	2.016	4	0.504	7.020	0.001 a
		Within groups	1.436	20	0.072		
		Total	3.452	24			

Abbreviation: ANOVA, analysis of variance.

a Statistically significant at 0.05 level of significance.

**Table 3:** Mean values, SD, and significance between tested group before and after thermocycling in relation to tested property

Repair gap/ concentration	Flexural strength		*p* -Value	Elastic modulus		*p* -Value	Impact strength		*p* -Value
	Before	After		Before	After		Before	After	
2.5 mm	68.9(1.1)	65.4(1.2)	0.002 *	3184.4(195.6) ^a^	2191.4(191.6)	0.002 *	2.3(0.3) ^a,b^	1.98(0.2) ^a,b,c^	0.071
2.5 mm–0.25%	80.8(0.5) ^a^ **	74.9(1.7) ^a^	0.000 *	3471.1(119.7) ^a,b,c,d^	3123.7(61.2) ^a,b^	0.000 *	2.2(0.3) ^a,c^	1.9(0.1) ^a,d,e^	0.21
2.5 mm–0.5%	75.2(0.9) ^b^	69.9(1.1) ^b^	0.000 *	3795.9(145.9) ^b,e,f^	3389.1(149.0) ^a,c,d^	0.002 *	2.8(0.5) ^b,c^	2.2(0.3) ^b,d,f^	0.055
0 mm–0.25%	81.9(1.3) ^a^	73.4(0.8) ^a,c^	0.000 *	3745.3(190.2) ^c,e,g^	3607.1(84.8) ^c,e^	0.176	3.5(0.4) ^d^	2.7(0.4)	0.016 *
0 mm–0.5%	76.1(1.4) ^b^	71.6(1.4) ^b,c^	0.001 *	3722.3(304.2) ^d,f,g^	3371.9(278.7) ^b,d,e^	0.094	3.8(0.3) ^d^	2.2(0.2) ^c,e,f^	0.000 *

Abbreviation: SD, standard deviation.

* Statistically significant at 0.05 level of significance. *p* -Value of *t* -test for thermocycling effect per gap and concentration horizontally.

** Same alphabets within each column showed statistically insignificant difference in mean.

Regarding the elastic modulus, lowest mean was found at 2.5 mm (control) before and after thermocycling. While the highest average was noticed at 2.5 mm-0.5% before thermocycling and at 0mm-0.25% after thermocycling, the elastic modulus of ND-reinforced groups was significantly increased when compared with control group ( *p* ˂ 0.001) before and after thermocycling except control versus 2.5 mm–0.25% before thermocycling ( *p* = 0.202). In between ND-reinforced groups without thermocycling effect, all pairs had statistically insignificant differences. While after thermocycling, the only pair that had significant difference was 2.5 mm–0.25% versus 0 mm–0.25% ( *p* = 0.002). Within the groups, elastic modulus values were reduced significantly after thermocycling except 0 mm–0.25% ( *p* = 0.176) and 0 mm–0.5% ( *p* = 0.094; Table 3 ).

The lowest impact strength values were found at 2.5 mm–0.25% before and after thermocycling. While the highest values were noticed at 0 mm–0.5% before thermocycling and at 0 mm–0.25% after thermocycling, ND-reinforced groups, 0 mm–0.25% and 0 mm–0.5%, showed significant increase in impact strength when compared with control group with *p* = 0.001 and *p* <0.001, respectively, before thermocycling. However, comparison between control and ND-reinforced group after thermocycling, statistically significant differences were found between control versus 0 mm–0.25% ( *p* = 0.000) and control versus 2.5 mm–0.5% ( *p* = 0.002). In between ND-reinforced groups, before thermocycling effect, insignificant differences were found at 2.5mm–0.25% versus 2.5mm–0.5% ( *p* = 0.105), 0 mm–0.25% versus 0mm–0.5% ( *p* = 0.595). However, after thermocycling, significant differences were found at 2.5mm–0.25% versus 0 mm–0.25% with *p* ˂0.001, 2.5 mm–0.25% versus 0 mm–0.5% with *p* ˂ 0.001. Comparing the effect of thermocycling on impact strength for each group showed a significant reduction only in 0 mm–0.25% ( *p* = 0.016) and 0 mm–0.5% ( *p* = 0.000) ( Table 3 ).

Combined effect of thermocycling and repair gap with ND concentration was analyzed through two-way ANOVA and test was run separately for each property ( Table 4 ). It was found that combined effect of thermocycling and repair gap had significant effect on each tested property. As shown in Table 5 , there were variations in nature of failure of 2.5mm unmodified repair gap showing mostly adhesive, followed by cohesive failure. While in ND repair groups, the dominant type of failure was adhesive, in addition to the absence of cohesive fracture and increase of mixed fracture especially with 0 mm repair gap. According to chi-squared test, no significant difference was found in fracture type before and after thermocycling.

**Table 4:** Two-way ANOVA for flexural strength, impact strength, and elastic modulus

Property	Source	Type III sum of squares	df	Mean square	F-Value	*p* -Value
Flexural strength	Gap concentration	769.685	4	192.421	133.744	0.000 a
	Thermocycling	382.317	1	382.317	265.732	0.000 a
	Gap concentration a thermocycling	34.564	4	8.641	6.006	0.001 a
	Error	57.549	40	1.439		
	Total	273802.326	50			
Elastic modulus	Gap concentration	6446992.927	4	1611748.232	46.034	0.000 a
	Thermocycling	2499946.385	1	2499946.385	71.402	0.000 a
	Gap concentration a thermocycling	1035742.863	4	258935.716	7.396	0.000 a
	Error	1400486.905	40	35012.173		
	Total	575939242.042	50			
Impact strength	Gap concentration	9.410	4	2.352	22.107	0.000 a
	Thermocycling	6.351	1	6.351	59.683	0.000 a
	Gap concentration a thermocycling	3.450	4	.863	8.105	0.000 a
	Error	4.257	40	.106		
	Total	348.388	50			

Abbreviation: ANOVA, analysis of variance.

a Statistically significant at 0.05 level of significance.

**Table 5:** Nature of failure of flexural strength specimens

Groups	Thermocycling	Nature of failure			*p* -Value
		Adhesive	Cohesive	Mixed	
2.5 mm	Before	6	3	1	0.89
	After	5	4	1	
2.5 mm–25%ND	Before	8	–	2	1.00
	After	9	–	1	
2.5 mm–0.5%ND	Before	9	–	1	1.00
	After	10	–	–	
0 mm–0.25%ND	Before	7	–	3	1.00
	After	6	–	4	
0 mm–0.5%ND	Before	7	–	3	1.00
	After	7	–	3	

Note: All *p* -values are statistically insignificant.

**Fig. 4:**
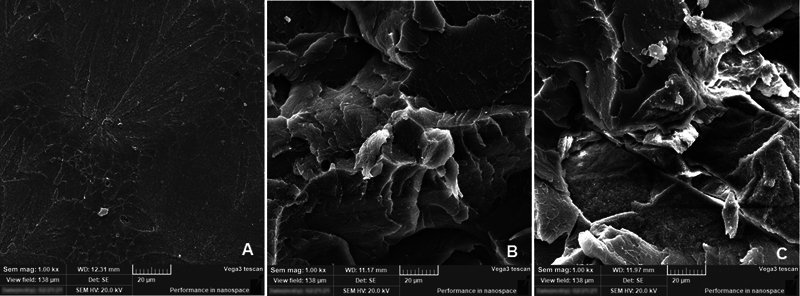
Representative scanning electron microscopy images for fracture surface of flexural strength test specimens 2.5 mm groups after thermocycling. ( **A** ) 2.5 mm–0%ND, ( **B** ) 2.5 mm–0.25%ND, and ( **C** ) 2.5 mm-0.5%ND.

**Fig. 5:**
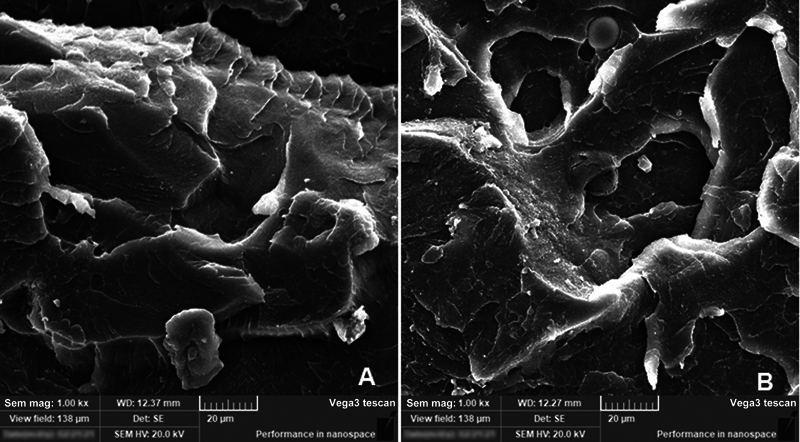
Representative scanning electron microscopy images for fracture surface of flexural strength test specimens 0 mm groups after thermocycling. ( **A** ) 0 mm–0.25%ND), ( **B** ) 0 mm–0.5%ND.

The representative micrographs were displayed at medium magnification of x1000 to show the important surface features of both 0– and 2.5 mm specimens after going through flexural strength tests ( Figs. 4 and 5 ). Fig. 4A showed a smooth surface represents a brittle fracture type for control specimen, while ND addition specimens (0.25% and 0.5% ND) showed different topographical features, the surface is rough with thicker lamellae ( Figs. 4B , 4C , and 5 ). With 0.25%ND addition, irregular FS with multiple sharp step lamellae indicated ductile fracture mode, in addition to the absence of clusters which indicates well distribution of nanoparticles within PMMA resin matrix ( Figs. 4B and 5A ). With increasing concentration of ND addition (0.5%ND), the FS showed same irregular and sharp lamella but with some small cluster formation of ND particles ( Figs. 4C and 5B ).

## Discussion

One of the improvements in denture repair is the repair resin reinforcement with nanoparticles. Previous studies have suggested ND addition to heat polymerized PMMA denture base resins. 18 19 22 However, no previous studies investigated its addition to repair resin along with different repair gap size. Therefore, this *in vitro* study aimed to assess the influence of ND addition combined with repair gap modifications on the flexural strength, elastic modulus, and impact strength of repaired denture base. The first and second null hypotheses of the present study were rejected since the size of repair gap and the addition of ND affected the repair strength and significantly improved the flexural strength, elastic modulus, and impact strength.

The continuous masticatory forces cause deformation of denture base material; therefore, high flexural strength is required to decrease the probability of denture fracture. 23 Among the factors that could affect the repair strength is the repair material reinforcement and/or surface design. 2 Depending on the results of the present study, the flexural strength increased in comparison to control group. This increase could be attributed to the well distribution of fine ND within resin matrix. 22 Furthermore, treated NDs have several reactive groups such as –COOH and –OH that improve the bond between NDs and resin matrix. Heat treatment of NDs was done to increase the interaction between ND and PMMA resin matrix (carbonyl groups) 20 and decrease particles agglomeration. 18

According to the finding of this study, the flexural strength decreased as ND concentration increased (0.5%). As the concentration increased above saturation limit, ND agglomerated forming clusters that affect the flexural strength of reinforced resin. 18 Al-Harbi et al 18 added 0.5, 1, and 1.5 wt% ND and reported an increased strength with 0.5% filler, while declined as ND concentration increased. Also, a previous study, 22 has found a significant increase in strength with the incorporation of 0.5 wt% ND. Accordingly, the recommended ND addition to denture base material in low concentrations is in agreement with the finding of the present study. Low ND showed higher flexural strength before and after thermocycling. This may be due to the saturation level at 0.25% and after that clusters were formed with increased concentrations up to 0.5%.

Repair gap decreasing up to 0 mm with 45 degrees beveling was suggested in previous study and observed that the repair strength improved with decrease in the repair gap and recommended 0 mm, 0.5 mm, or 1.0 mm with beveled repair surface design. 5 Therefore, 0 mm repair gap was selected for comparison in the present study. Based on the results of present study, although 0 mm substantially improved the flexural strength as compared with control, the repair gap did not show significant differences per respective concentrations in disagreement with a prior study that found remarkable improvement of flexural strength at 0 mm repair gap. 5 The differences in results may be related to the presence of ND as reinforcement for repair resin that may have changed the behavior of material during failure.

In concern thermal stress effect, the flexural strength decreased significantly after thermocycling and there was a significant decrease per respective concentrations and repair gap. A previous study showed similar results of 23 substantial reduction in flexural strength following thermal stressing. This effect may be attributed to water sorption, that results in. 24 25 water molecules occupying inter-polymeric gap and forcing the polymer chains apart. 26 27 In addition to the plasticizing effect of absorbed water that allows the chains to slide easily under load, affecting the mechanical properties of resin material. 28 29 Intraorally, moisture and thermal fluctuation accelerate water absorption. 25 Water absorption rate is affected by temperature 28 therefore, thermal stress may enhance water entrance into the polymer mass decreasing the polymer's strength after thermal cycling specially at repair/resin interface. 24 29

In the nonreinforced group, adhesive failure was common before and after thermocycling, while ND-reinforced groups showed more adhesive and disappearance of cohesive fractures exhibited strong repair material and weakness at repair/resin interface. The nature of failure was closely the same after thermocycling per respective concentrations and repair gap, where the mixed failure was appeared with 0 mm repair gap. This finding proves the influence of thermal stress on the repair bond strength at the resin/repair interface and thin edge of 0 mm gap displayed mixed failure. In agreement with previous study, 28 reported more adhesive failures of repaired denture base after thermocycling.

Elastic modulus affects material rigidity, as it increases in the value, the elastic deformation decreases, thus the material would be more rigid. 31 A denture base material with high elastic modulus can resist permanent deformation c caused by constant stress or strain during mastication, 31 32 The results showed increase of, the elastic modulus with 0.5% NDs at 2.5 mm and 0.25% and 0.5%NDs at 0 mm compared to control group and above the minimum recommended value (2000 MPa) by ADA specifications. 33 The probable cause for the increased elastic modulus could be explained based on the homogenous distribution and micro-size distance between NDs minimizing the polymer chain immobilization effect. 31 32 Another advantage of decreasing repair gap is reducing the amount of autopolymerized repair resin and subsequently its drawbacks. 5 When comparing elastic modulus of 0 mm with 2.5 mm after thermocycling, 2.5 mm groups significantly decreased with no effect on 0 mm group. This may be related to increased amount of repair material and effect of water sorption and thermal stress on the increase amount of repair resin in comparison to 0 mm repair gap. 5

The main cause of denture fracture is accidental drop that may occur during denture cleaning, coughing, sneezing, or sudden strokes to the denture. 34 Accordingly, adequate impact strength is important for denture base resin to resist denture fracture and improve its durability. 18 Charpy's impact test was chosen for this study in which v-shaped notches were made in the specimens resembling denture frenal notch to act as stress concentration area. 10 This was confirmed by the nature of failure of impact specimens where all specimens displayed cohesive fracture type at the v-notch.

The results revealed that the addition of ND did not alter the impact strength in 2.5 mm groups. This finding was coincidence with previous study which found that 0.5%ND did not affect the impact strength off denture base resin. 5 In contrast, 0 mm groups increased the impact strength of repaired specimens. As the concentration was same for both gaps, this effect may be due to the decreased amount of repair resin and this was proven by thermal stress effects where 0 mm groups showed increased impact strength even after thermocycling. Comparing thermocycling effect per respective gap and concentrations, the impact strength was decreased and this may be attributed to the aforementioned effect of thermal stress and water sorption on PMMA resin material.

From the clinical point of view, repair resin could be modified with low ND concentrations. This addition significantly improved the repair strength combined with 0 mm repair gap that considered a positive effect with ND addition. However, the results of this study could be interpreted with cautions before clinical applicability due to the limitation of this study. These limitations included using one brand of denture base resin and repair resin, and specimens were not simulating denture configurations. Moreover, *in vitro* study lacks oral conditions such as saliva and masticatory forces. Therefore, further studies on different repair resin material with low ND concentrations in conditions simulating oral environments are required.

## Conclusions

The following conclusions can be drawn:

1. Incorporating 0.25% and 0.5% ND into autopolymerized repair resin substantially improved the flexural strength, elastic modulus, and impact strength of repaired denture base resin.
2. 0 mm repair gap had no influence on flexural strength while elastic modulus and impact strength were significantly improved.
3. Thermocycling adversely affected the repair strength with ND addition combined with repair gap modifications.
